# Poisson-Nernst-Planck Models of Nonequilibrium Ion Electrodiffusion through a Protegrin Transmembrane Pore

**DOI:** 10.1371/journal.pcbi.1000277

**Published:** 2009-01-30

**Authors:** Dan S. Bolintineanu, Abdallah Sayyed-Ahmad, H. Ted Davis, Yiannis N. Kaznessis

**Affiliations:** Department of Chemical Engineering and Materials Science, University of Minnesota, Minneapolis, Minnesota, United States of America; University of Houston, United States of America

## Abstract

Protegrin peptides are potent antimicrobial agents believed to act against a variety of pathogens by forming nonselective transmembrane pores in the bacterial cell membrane. We have employed 3D Poisson-Nernst-Planck (PNP) calculations to determine the steady-state ion conduction characteristics of such pores at applied voltages in the range of −100 to +100 mV in 0.1 M KCl bath solutions. We have tested a variety of pore structures extracted from molecular dynamics (MD) simulations based on an experimentally proposed octomeric pore structure. The computed single-channel conductance values were in the range of 290–680 pS. Better agreement with the experimental range of 40–360 pS was obtained using structures from the last 40 ns of the MD simulation, where conductance values range from 280 to 430 pS. We observed no significant variation of the conductance with applied voltage in any of the structures that we tested, suggesting that the voltage dependence observed experimentally is a result of voltage-dependent channel formation rather than an inherent feature of the open pore structure. We have found the pore to be highly selective for anions, with anionic to cationic current ratios (I_Cl−_/I_K+_) on the order of 10^3^. This is consistent with the highly cationic nature of the pore but surprisingly in disagreement with the experimental finding of only slight anionic selectivity. We have additionally tested the sensitivity of our PNP model to several parameters and found the ion diffusion coefficients to have a significant influence on conductance characteristics. The best agreement with experimental data was obtained using a diffusion coefficient for each ion set to 10% of the bulk literature value everywhere inside the channel, a scaling used by several other studies employing PNP calculations. Overall, this work presents a useful link between previous work focused on the structure of protegrin pores and experimental efforts aimed at investigating their conductance characteristics.

## Author Summary

Protegrins are small peptides with strong antimicrobial properties, believed to kill bacteria primarily by forming nonselective pores in the bacterial membrane. This nonspecific and highly effective mechanism of action has created significant excitement about the use of protegrins as therapeutic antibiotics. However, a lack of understanding of the fundamental processes that lead to pore formation and bacterial death has proven to be a major bottleneck in the rational design of protegrin-based antibiotics. In the present work, we have carried out computational investigations of the diffusion of ions through a protegrin pore. We have thereby provided a connection between previous experimental and simulation work aimed at elucidating the structure of the protegrin pore and earlier experimental work investigating the ion transport characteristics of protegrin pores. The ion diffusion characteristics of protegrin pores are likely to be important in their ability to kill bacteria, as the uncontrolled flow of ions through a bacterial membrane will result in membrane depolarization and the loss of vital membrane functions. The present work thus represents an important first step in modeling and quantifying the timeline of events that lead to the killing of bacteria by protegrins. Furthermore, the computational tools that we have presented herein are easily extendible to similar systems, in particular other antimicrobial peptides.

## Introduction

Antimicrobial peptides (AMPs) are small proteins produced by the innate immune system of many plants and animals as a first line of defense against bacterial infections [1],[2]. Due to their persistence in nature as well as their nonspecific mechanism of action, there has been significant research activity aimed at designing novel antibiotics based on AMPs [3]; the expectation is that bacteria will not develop significant resistance to antibiotics designed based on these peptides. Thus far, such drug design efforts have been largely hampered by a lack of understanding of the fundamental mechanism of action of AMPs. Although recent evidence suggests that intracellular targets may play an important role in the action of many AMPs [2], [4]–[7], there is a strong body of evidence suggesting that the ability of peptides to interact with and disrupt the bacterial membrane is essential to their mechanism of action [1], [8]–[10]. AMPs of various structural classes have been shown to have significant disruptive effects on both living bacterial membranes and model membrane systems, such as lipid bilayers [8],[11],[12] and lipid monolayers [8],[13],[14]. For thorough reviews of several proposed mechanisms of membrane disruption, the reader is referred to [10]. Most relevant for the present work is the model in which AMPs aggregate to form large, nonselective pores in the bacterial membrane, which result in uncontrolled ion leakage, decay of the transmembrane potential, uncontrolled water transport, loss of cell contents and ultimately cell death. We have focused our efforts on protegrin-1, a particularly potent antimicrobial peptide, which has recently been shown to form such pores in lipid bilayers of certain compositions.

**Figure 1:**
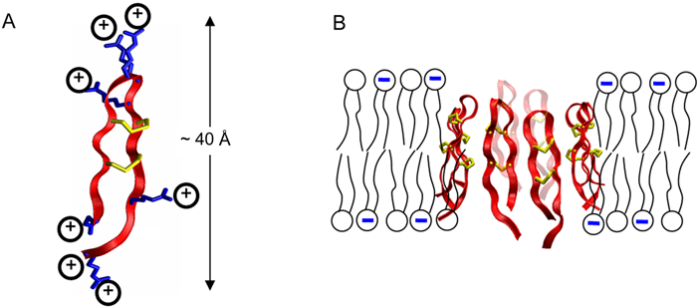
Pore-forming protegrins. (A) A single protegrin peptide; note the abundance of positive charges due to arginine side chains (shown in blue). Cysteine-cysteine disulfide bonds are shown in yellow. (B) Eight protegrin peptides aggregate to form a membrane-spanning pore.

Protegrins are small peptides isolated from porcine leukocytes that exhibit strong antimicrobial activity against a broad range of both Gram-positive and Gram-negative bacteria [15]. Protegrins are characterized by a β-hairpin conformation that is held together by two cysteine-cysteine disulfide bonds. They contain 16–18 amino acids, and are typically highly cationic (charge of +7), with the positive charges arising from arginine residues at the hairpin turn region and the two termini. In the present work, we focus on the most prevalent natural form of protegrin, designated as PG-1, with the amino acid sequence RGGRLCYCRRRFCVCVGR-NH_2_ (PDB entry 1pg1). Mani and coworkers [16] have conducted solid-state NMR experiments to investigate the membrane-bound structure of a PG-1 peptide, and have concluded that this peptide likely forms octomeric pores in lipid bilayers composed of a 3∶1 mixture of palmitoyloleoyl-phosphatidylethanolamine (POPE) to palmitoyloleoyl-phosphatidylglycerol (POPG) [16]. Langham and coworkers used the structure suggested from these NMR experiments as the starting configuration of a molecular dynamics simulation in a lipid bilayer of the same composition [17]. This simulation showed the pore to be stable over more than 150 ns. Figure 1 shows a cartoon representation a single protegrin peptide, as well as a side view of the proposed pore structure.

Prior to these studies of the pore structure of protegrin, early evidence of protegrin pores was provided by the experiments of Mangoni and coworkers [18] and Sokolov and coworkers [19], in which the conductance characteristics of protegrin-treated membranes were measured. Such knowledge of the nonequlibrium ion flow through protegrin pores may be closely related to their mechanism of action, since the unrestricted flow of ions through the membrane could result in potentially lethal membrane depolarization. Mangoni and coworkers conducted voltage clamp experiments in *Xenopus laevis* oocyte membranes treated with protegrin-1 and several analogues [18]. They found that protegrins form weakly anion selective pores in the presence of several different salts, with KCl solutions exhibiting almost no selectivity. Furthermore, they found that the conductance of such pores does not exhibit any voltage dependence over a voltage range of −100 to +30 mV. Sokolov and coworkers [19] carried out conductance measurements across several different types of planar phospholipid bilayers treated with protegrin-1 as well as protegrin-3 (PG3, amino RGGGLCYCRRRFCVCVGR; note the only difference between PG-1 and PG-3 is the substitution of a glycine residue at the third position in PG-3 instead of the arginine residue in PG-1). These authors found that both protegrin analogues form weakly anion selective channels in mixed phospholipid bilayers, and moderately cation-selective channels in bilayers containing negatively charged bacterial lipopolysaccharide (LPS). They reported a voltage-dependent single-channel conductance in the range of 40–360 picoSiemens (pS), depending on the peptide used, the lipid bilayer composition, and the applied voltage.

In the present work, we attempt to explain and quantify the conductance behaviour of protegrin pores in terms of the structural information from NMR experiments [16] and molecular dynamics simulations [17]. In particular, we explore the connection between structural features such as the size of the pore opening and the magnitude of the conductance, as well as the surprising experimental finding of both Mangoni and coworkers [18] and Sokolov and coworkers [19] that the protegrin pore is only slightly anion selective, despite having a total charge of +56. Our investigation is based on the Poisson-Nernst-Planck (PNP) theory, a continuum method of calculating non-equilibrium ion concentrations and fluxes around a fixed structure in the presence of an applied electrical voltage. In our model, we simulate a voltage across a protegrin pore embedded in a lipid bilayer patch, and measure the resulting current. Since the PNP model requires a rigid structure, we perform the calculations using several snapshots from the MD simulations of Langham and coworkers [17]. The description of the underlying equations and the numerical scheme used to solve them is deferred to the Methods section below.

## Results

**Figure 2:**
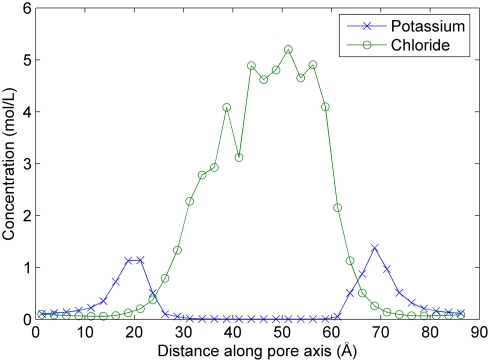
Concentration profiles of potassium and chloride. The values shown are averages over the x and y directions within the ion-accessible region. These profiles correspond to the PNP model applied to a snapshot at 93.5 ns of the NPT segment of the MD simulations of Langham et al., with an applied voltage of −20 mV, 0.1 M KCl bath concentrations, and diffusion coefficient profile D4.

In all of the results presented herein, we have modeled electrodiffusion of ions through a protegrin pore bathed in KCl solutions under a constant applied voltage. The solution of the PNP equations yields the nonequilibrium concentration profiles of both ions and the electrostatic potential profile. Figure 2 shows a plot of the concentration profiles of both potassium and chloride ions as a function of location along the pore axis for typical model parameters. The values shown represent average concentrations in small planar slabs along the pore axis.

From the concentration and electrostatic potential profiles, the net current can be obtained using equation 7 (see Methods section). Repeating this for multiple voltage values yields a current-voltage (I–V) relationship that can be compared to experimental results.

**Figure 3:**
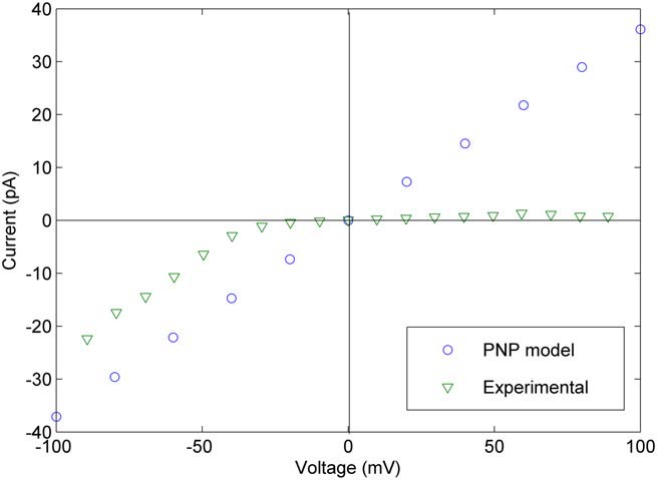
Comparison of I–V curves from the PNP model and experiments [19]. All data correspond to a snapshot at 93.5 ns of the NPT segment of the simulations of Langham and coworkers [17], using diffusion coefficient profile D4 (see Methods section). KCl salt bath concentrations were set to 0.1 M on both sides of the membrane in order to match experimental conditions.

Figure 3 shows the I–V curve obtained for a snapshot at 93.5 ns of the NPT segment of the MD simulations of Langham and coworkers [17], along with experimental data from Sokolov and coworkers [19]. In both cases, these data correspond to 100 mM KCl solutions on both sides of the lipid bilayer.

The data in Figure 3 were obtained by setting the diffusion coefficient to 10% of its bulk value everywhere inside the channel, which represents an empirically adjusted value used to partially account for the approximate nature of PNP theory. The sensitivity of the calculations to the choice of diffusion coefficient is discussed below. The corresponding conductance (the slope of the line in Figure 3) is approximately 280 pS over the entire voltage range, in agreement with the experimental range of 40–360 pS [19]. The value of the voltage in our model corresponds to the side nearest to the protegrin termini, while virtual ground is defined on the side of the hairpin turns (refer to Figure 1).

Table 1 shows the conductance characteristics obtained for several simulated structures, along with dimensions that characterize these structures. The nanosecond values in the structure description column indicate the simulation time from the start of the constant pressure and temperature (NPT) segment of the MD simulations of Langham and coworkers [17]. All data except the reversal potential correspond to a symmetric 100 mM KCl solution with an applied voltage of −20 mV.

**Table 1:** Conductance characteristics measured for a variety of structures.

Structure Description	Effective Diameter of Narrowest Constriction* (Å)	Average Effective Diameter of Channel (Å)	Total Conductance (pS)	Current Ratio (I_Cl−_/I_K+_) at −20 mV	Reversal Potential in a 10∶1 KCl Gradient
60 ns	11.3	28.9	427	374	−57.4
65 ns	7.51	26.9	361	2640	−60.7
70 ns	6.36	27.0	384	2660	−49.8
80 ns	5.97	23.7	286	6860	−61.4
81.5 ns	10.0	25.2	405	585	−60.2
85.5 ns	7.92	24.6	361	953	−60.1
87.5 ns	8.11	25.8	364	731	−59.0
91.5 ns	7.80	25.4	387	564	−58.7
93.5 ns	9.44	24.4	367	338	−58.7
NMR structure proposed by Mani et al [16]	14.5	31.5	679	389	−57.8
Toroidal pore after minimization and heating	12.8	33.2	664	330	−59.2
Toroidal pore after 50 ns of NP_z_AT simulation	12.7	35.8	479	333	−56.6

*: Effective diameters are based on a grid search of the ion-accessible area, as determined using a probe radius of 1.4 Å (see Methods section for further details).

We also analyzed the sensitivity of the results to several model parameters, and found the diffusion coefficient profile to have the most significant effect. Four diffusion coefficient profiles were used, shown in Figure 3 in the Methods section below. The resulting conductance characteristics obtained using the pore structure at 93.5 ns are tabulated in Table 2. The rationale for each diffusion coefficient profile is discussed in greater detail in the methods section.

**Table 2:** Conductance characteristics as a function of diffusion coefficient profile.

Diffusion Coefficient Profile	Total Conductance (pS)	Current Ratio (I_Cl−_/I_K+_) at −20 mV
D1 - bulk values everywhere	2310	220
D2 - scaled from bulk values as per MD simulation results	1550	314
D3 - hydrodynamic model based on channel radius profile (based on [27],[28])	1280	242
D4 - 10% of bulk values everywhere inside the channel	367	564

All data correspond to the snapshot at 80 ns of the MD simulation.

In order to quantify the relative importance of certain charged moieties within the structure, we analyzed the effects of removing the positive charges of the arginine residues of the peptides in one case, the negative charges in the POPG lipids in another case, and both in a third case, all the while keeping the rest of the system unchanged. The results are summarized in Table 3.

**Table 3:** Effects of various charged moieties in the system on conductance behaviour.

Model	Total Conductance (pS)	Current Ratio (I_Cl−_/I_K+_) at −20 mV
All charges present	286	6860
Positive arginine charges removed	34.5	0.030
Negative POPG charges removed	328	8480
Both positive arginine charges and negative POPG charges removed	16.5	0.17

All values correspond to the simulation snapshot at 80 ns, using diffusion coefficient profile D4.

## Discussion

The concentration profiles obtained from solving the PNP equations for a typical simulation snapshot are shown in Figure 2. Due to the high concentration of positive charge in the pore structure, the concentration ions of potassium ions inside the pore is extremely low, while the concentration of chloride is extremely high in order to neutralize the arginine charges. Although concentration values as high as 5 mol/L exceed the saturation concentration of KCl, one must keep in mind that these are only local values in small regions, and cannot be strictly interpreted as concentrations in the context of solution thermodynamics. The Nernst-Planck equation can be alternatively cast as a Smoluchowski-type equation, in which case the concentrations represent relative probabilities of finding an ion at a certain location, which is perhaps a more relevant way to interpret these results. However, such high concentrations may also approach the limit of the applicability of PNP theory, in particular with regards to the lack of direct ion-ion correlations and ion-ion van der Waals interactions. The effects of such inaccuracies cannot be easily ascertained. However, previous work [20] criticized the accuracy of PNP models in narrow channels due to the overestimation of screening effects that arises from the continuum approximation, in which an ion is locally screened by its counterion, regardless of geometric restrictions on the presence of both ions. In the case of a pore as highly charged as the protegrin pore, where the concentration of chloride inside the pore is orders of magnitude higher than potassium, such overestimations of screening effects would not arise. Corry and coworkers [20] indeed found that the accuracy of PNP models improved in moderately charged pores. In our system, the extent to which inaccuracies arising from the lack of ion-ion correlations in PNP models are offset by improvements in accuracy that arise from the presence of only the chloride ion in the channel is not clear. This should be kept in mind as a possible caveat to the applicability of PNP theory to highly charged channels in the discussions that follow. It should also be noted that PNP theory is the only relevant continuum theoretical formulation for systems like the one investigated here, with microscopic electrodiffusion phenomena at molecular scales.

The current-voltage relationship obtained by solving the PNP equations for a snapshot corresponding to 93.5 ns into the NPT segment of the MD simulations of Langham and coworkers is shown in Figure 3. Also shown is the experimental I–V curve reported by Sokolov and coworkers in 100 mM KCl symmetric salt baths. The most notable difference in this figure is the lack of voltage-dependent behaviour in our model, whereas the experimental data show a clear increase in conductance at negative voltages. The sign of the voltage in the experiments of Sokolov et al refers to the voltage value on the side opposite (*trans*) of the solution containing protegrin; in our model, the value of the voltage corresponds to the side with the protegrin termini, while virtual ground is defined on the side of the hairpin turns.

The strong voltage dependence observed experimentally in Sokolov et al [19] is most likely attributable to major conformational changes of the channel caused by the applied voltage, rather than asymmetry in ion flux due to inherent pore structure asymmetry. At negative *trans* voltages, the channel is likely formed and open, while at low negative voltages and positive *trans* voltages, the channel does not form. This behavior cannot be captured in the PNP model, which uses the same rigid pore structure at all voltages (for a given snapshot). Given the good agreement between the conductance at negative voltages obtained in the simulation and that in the experiments, we propose that the pore structure used here corresponds closely to the open pore structure formed at negative voltages in the experiments. As mentioned earlier, Mangoni and coworkers [18] found no voltage dependence of the conductance in their experiments with *Xenopus laevis* oocyte membranes treated with PG-1, which supports the hypothesis that the voltage-dependence is a function of membrane composition and the ability of protegrins to form conductive pores, rather than an inherent characteristic of the pore structure.

Sokolov and coworkers [19] report single-channel conductance values in the range of 50–100 pS for PG-1 in a black lipid bilayer, 40–100 pS for PG-3 in the same composition bilayer, and 80–360 pS for PG-3 in bilayers containing either lipopolysaccharide (LPS) or Lipid A, both of which are anionic lipids. The conductance presented above (the slope of the I–V curve in Figure 3) is around 367 pS, well within the range of the experimental values observed by Sokolov and coworkers in their anionic bilayers. Even considering the limitations and typical overestimations that sometimes arise from the use of PNP theory, the lower conductance values of 50–100 pS observed in neutral black lipid bilayers do not seem to be compatible with the structure extracted from the equilibrated MD simulations of Langham and coworkers which were conducted in a 3∶1 POPE∶POPG lipid bilayer [17] (and even less compatible with the structure directly proposed by the NMR experiments of Mani and coworkers [16], as discussed below).

From the experimental evidence discussed, it is clear that the exact membrane composition strongly affects the ability of protegrins to form pores, and likely the nature of the pores themselves. However, for anionic membrane compositions, our data along with the experimental observations discussed seem to suggest that pores are similar in structure across different types of anionic bilayers. This would explain why the pore structure from the equilibrium simulations in reference [17] in a 3∶1 POPE∶POPG bilayer yields a conductance comparable (within the limits of PNP theory) to what was measured by Sokolov and coworkers at a negative voltage in negatively charged lipid bilayers composed of black lipids and LPS. Our data suggest that the low conductance values of 50–100 pS observed by Sokolov and coworkers in neutral black lipid membranes likely correspond to an altogether different pore structure, or a different conduction mechanism. The presence of anionic lipids could affect not only the ability of protegrins to form pores, but the types of pores formed, as well as having significant direct effects on ion permeation characteristics. We explore this last point in more detail below by repeating the calculations with the lipid charges turned off.

Perhaps the most significant and surprising difference between the experimental findings of Sokolov and coworkers [19] and the results of our model lies in the selectivity of the pore. The anionic current to cationic current ratios (I_Cl−_/I_K+_) in our simulations are typically on the order of 10^2^–10^3^, indicating a very strong anion selectivity of the protegrin pore. However, Sokolov and coworkers [19] found that protegrins generally form channels in black lipid membranes that are only ‘weakly anion-selective’. The addition of LPS to membranes treated with PG-3 resulted in membranes that were moderately cation-selective. In black lipid membranes, Sokolov and coworkers have reported that the reversal potential in a 10∶1 KCl gradient is on the order of 40 mV, which corresponds to a permeability ratio of P_Cl−_/P_K+_ of around 10. In order to facilitate an easier comparison, we carried out simulations with a 10∶1 (1.0 M to 0.1 M) KCl bath concentration ratio, and found the reversal potential to be between −49.8 and −61.4 mV, depending on the structure tested. From the Goldman-Hodgin-Katz equation [21], this corresponds to a permeability ratio of P_Cl−_/P_K+_ in the range of 18 to 3200:
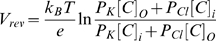
(1)Where [*C*]*_i_* and [*C*]*_O_* represent the concentrations of KCl in the baths surrounding the membrane, and P_K_ and P_Cl_ represent the permeabilities of K^+^ and Cl^−^, respectively. This can be re-arranged to solve for the permeability ratio as a function of the salt concentration gradient and the reversal voltage:
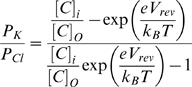
(2)


The high anion selectivity that we obtain from our model reflects the intuitive notion that a highly cationic protegrin pore would almost completely reject cations (with a charge of +7 on each peptide, the total charge of the protegrin pore is +56). The high anion selectivity is also corroborated by the simulations of Langham and coworkers [17], where only two events of sodium ions crossing the pore are observed in 150 ns, as compared to 55 events of chloride ions crossing (similar data were obtained for a simulation with KCl; unpublished results). This discrepancy between the PNP model and the experimental data cannot be explained simply based on differences in membrane composition. The much higher anion selectivity in our model may be due to a shortcoming of PNP theory in highly charged channels. However, it might also be the case that the lipids, rather than being almost aligned with the pore axis as they are in our model, orient in a perpendicular fashion to line the pore and form what is known as a toroidal pore. Recent NMR experiments suggest that this might indeed be the case [22]–[24]. In a toroidal pore, there would be a much higher likelihood of arginine sidechains forming persistent guanidinium-phosphate bonds with the surrounding lipid phosphate groups, which would partially neutralize the charged arginine moieties. To test this scenario, we also solved the PNP system for two snapshots from an MD simulation of a toroidal pore (unpublished results). As shown in Table 1, the high anion selectivity persists even in the toroidal pore models, ruling out this possible explanation for the discrepancy in selectivity.

### Effects of Variations in Pore Structure

The results of the PNP model solved for various structures are summarized in Table 1. None of the structures described showed any significant voltage-dependence in an applied voltage range of +/−100 mV (data not shown). The conductance values obtained based on structures extracted from the molecular dynamics simulations are all in good agreement with the experimentally observed values in anionic lipid bilayers (60–360 pS). The diameter of the smallest channel constriction in these structures ranges from 6–10 Å, which is on the lower end of the applicability of PNP modeling (see discussion below), but nonetheless sufficiently large to yield meaningful results. The variations that exist among different snapshots are the results of minor conformational changes caused by thermal fluctuations, in particular the motion of side chains into and out of the pore opening. Although these effects are noticeable, they are not drastic, and not as large as the differences between the MD structures from 60–93.5 ns and the other three structures. It is also worth noting that the diameter of the narrowest constriction, while it does somewhat correlate with conductance, is not the sole factor influencing it. For instance, the snapshot at 93.5 ns has a larger constriction diameter than either the 85.5 or 91.5 ns snapshots by almost 2 Å, and yet shows the same conductance as the 85.5 ns snapshot, and a lower conductance than the 91.5 ns snapshot. Thus, while steric effects clearly affect ion permeation, there are other conformational changes that have significant influence on pore conductance. These are likely electrostatic effects that arise from conformational changes of the charged side chains (e.g. arginine). These are explored in further detail below.

The results for the last three structures shown in Table 1 correspond most closely to the structure proposed by Mani and coworkers [16], which was used to start the MD simulations. This structure has a larger opening than the molecular dynamics simulations, and yields much higher conductance values. Even considering the inaccuracies in our model, these values are not in good agreement with experimentally measured values, which may suggest that the corresponding structures are not the dominant conformers. Since the NMR experiments do not directly resolve the positions of all side chains, we posit that the narrower opening observed in molecular dynamics simulations, which is primarily due to the motion of side chains into the interior of the pore, represents a more realistic structure. However, as discussed below, there are several other considerations that can significantly affect the conductance values obtained from PNP modeling.

### Specific Interactions of Arginine Side Chains

**Figure 4:**
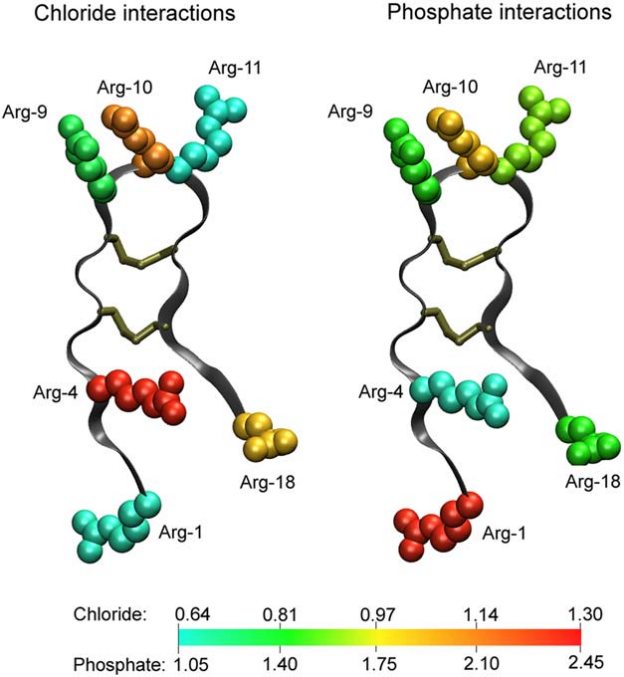
Interaction of arginine residues with phosphate groups and chloride ions. The numbers on the colour scales correspond to the number of phosphate groups/chloride ions within 7.5 Å of the ζ-carbon of the respective arginine residue. Data are averaged over the last 50 ns of the molecular dynamics simulation, as well as over the eight peptides.

In order to explain the conductance behavior observed in our model in terms of more specific structural features, we have analyzed the molecular dynamics simulations in greater detail. The high selectivity of the pore for anions that we observe in PNP calculations is likely due to electrostatic attraction between the positively charged guanidinium groups in the arginine side chains and negatively charged chloride ions (this is compellingly confirmed in the section ‘*Influence of embedded charges*’ below). However, competing with this interaction is the attraction of arginine side chains to negatively charged phosphate groups in the lipid head groups. In order to investigate the relative importance of these interactions, we have computed radial distribution functions for both chloride and phosphate groups from the ζ-carbon of all arginine side chains. For brevity, the resulting 48 plots are included as online supporting information (Text S1). Due to the axial symmetry of the pore and the equivalence of all eight peptides (except perhaps with respect to dimer pairings), we confine our discussion to averaged data for each arginine position. Figure 4 shows the number of chloride and phosphate atoms within 7.5 Å of the arginine ζ-carbon at each position, averaged over time as well as over all eight peptides. The value of 7.5 Å was selected to include the first large peak in all of the radial distribution functions (see Text S1).

As shown, arginine side chains at all positions interact fairly strongly with both phosphate groups and chloride ions. Arginine at position 1 exhibits a much stronger interaction with phosphate groups, indicated by an average value of 2.45 phosphate groups, as compared to 0.64 chloride ions. Also noteworthy is the difference at position 11, where phosphate interactions are again significantly stronger than chloride interactions. Although it may appear based on the averaged data that interactions with phosphate groups do not generally exclude interactions with chloride ions, the individual radial distribution functions (Text S1) show that in many cases, strong interactions of a particular residue with phosphate lead to weak interactions of that residue with chloride, and vice versa. However, considering the equivalence of all eight peptides, it is the averaged data that are more significant; the individual residue data in this case only reflect the fact that binding of particular arginine residues to phosphate or chloride ions often persists on the time scale of the molecular dynamics simulations.

Another notable feature in Figure 4 is the stronger interaction of arginine at position 4 with chloride as compared to the other arginine residues. Although the difference is not drastic (1.31 chloride ions for position 4 compared to 0.64 for position 1, or 1.14 for position 10), this may represent a significant electrostatic feature of the protegrin pore, particularly considering that the data represent an average over all eight peptides. The arginine side chain at position 4 is in a favorable location to be interacting with the aqueous pore interior, where it can have the largest effects on conduction characteristics. Considering the location of the other arginine residues near the termini and the β-sheet turn, as well as the discussion of phosphate interactions in the preceding paragraph, one could hypothesize that the purpose of arginine at position 4 is primarily to attract anions through the pore. In contrast, the remaining arginine side chains interact more strongly with lipid head groups in order to facilitate peptide insertion and stabilize the pore structure. This is consistent with the fact that arginine residues at position 4 have the weakest interactions with phosphate groups. We investigate this hypothesis in further detail in the section ‘*Influence of embedded charges*’ below.

**Figure 5:**
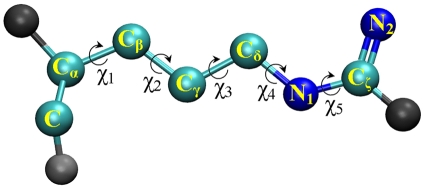
Definition of arginine side chain dihedral angles.

Additionally, we have also investigated the rotameric conformations of all arginine side chains in order to confirm that they are indeed well-sampled and found in reasonable rotameric states throughout the relevant portion of the molecular dynamics simulations. Figure 5 shows the definition of the side chain dihedral angles that we have used.

We have included plots of all dihedral angles for all arginine side chains as online supporting information (Text S1). Plots are labeled as “peptide-position” - for example, plot P1-6 corresponds to peptide 1, position 6. Dihedral angle χ_1_ generally occurs in the energetically favorable *trans* conformation (values near ±180°), but also occasionally in a *gauche* conformation (values near ±60°), as in P4-1 to P4-18, and P8-1 to P8-9. Dihedral angle χ_2_ is largely found in the trans conformation, with only a handful of noticeable exceptions in the *gauche* conformations (P2-10, P3-9,10,11, P6-9, P7-18 and P8-9). The same general trend of preference for trans conformations is true for angles χ_3_ and χ_4_, with χ_4_ (blue) showing perhaps the greatest flexibility (see, for instance, the variation in P1-1,18; P3-1,10,11; P7-1; P8-18). Angle χ_5_ is exclusively found in an eclipsed conformation (value near 0°), shown by the yellow bands in the centres of all plots. This is not surprising, considering the partial charge in the CHARMM27 force field is −0.47 for N_1_ and +0.64 for C_ζ_, resulting in a strong attraction between these atoms (refer to Figure 5 above for atom labels). Overall, while some variation occurs due to thermal fluctuations, the arginine side chains are largely found in common rotameric states.

### Effects of the Diffusion Coefficient Profiles

**Figure 6:**
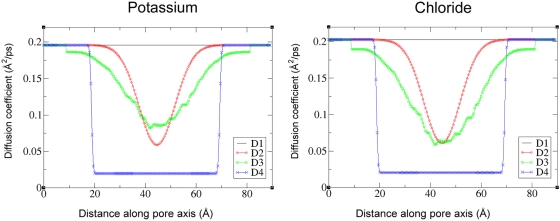
Ion diffusion coefficient profiles. All the profiles tested (D1–D4) are shown for both ions. Profile D3 is the only one that varies with the pore structure; the figure depicts diffusion coefficient profiles corresponding to the structure at 93.5 ns into the NPT segment of the MD simulations.

One of the most important parameters required for successful PNP modeling is the space-dependent diffusion coefficient of all diffusing ionic species. The diffusion coefficient is often empirically adjusted to account for the approximate nature of the PNP theory. The value of the diffusion coefficient is expected to be different from the bulk literature values in the constricted molecular geometry of the pore interior [25],[26]. Unfortunately, the diffusion coefficients inside the pore are also one of the most difficult parameters to assign. As far as we are aware, no experimental techniques are capable of measuring the diffusion coefficient of ions within a molecular pore to a high accuracy and with sufficiently high spatial resolution; certainly no such data are available for the protegrin pore. We have therefore used four different empirically-derived diffusion coefficient profiles, denoted as D1–D4. Plots of the diffusion coefficient of potassium and chloride as a function of the pore axial coordinate for each profile are shown in Figure 6 in the Methods section below.

In all cases, the diffusion coefficient was assumed to be isotropic and invariant in the x and y directions (in the plane of the bilayer). The PNP system was solved for all four diffusion coefficient profiles (D1–D4) using the structure at 80 ns of the MD simulation. The results are summarized in Table 2, along with a brief description of each profile. None of the cases tested showed any significant voltage-dependence for applied voltages in the range of +/−100 mV.

As already mentioned, the experimentally reported conductance is 50–100 pS in black lipid membranes and 60–360 pS in anionic membranes [19]. Clearly, diffusion coefficient profiles D1 and D2 result in unacceptably high conductance values, indicating that the diffusion coefficient inside the channel should be significantly lower. Using the hydrodynamic model of [27], which was also employed successfully in references [28],[29] in PNP models of α-hemolysin channels, yields a slightly lower value, but still outside of the range of the experimentally determined conductance. The diffusion coefficient profile D4 yields the best agreement with experimental conductance data. Although setting the diffusion coefficient to 10% of the bulk value may seem a low estimate, similar scaling was used by Furini and coworkers [30] and Kurnikova and coworkers [31] in successfully modeling KcsA channels and Gramicidin A channels, respectively. Considering the differences between our model and the experimental systems, variations due to minor structure fluctuations, as well as the approximate nature of our model, profile D4 appears to be a reasonable estimate for the diffusion coefficients, and can be treated as an effective fitting parameter.

### Influence of Embedded Charges

As shown in Table 3, the positively charged arginine residues, which impart the pore a total charge of +56, are by far the dominant factor influencing conductance behaviour. Removal of the arginine charges decreases conductance by a full order of magnitude, and changes the selectivity of the pore from strongly anionic to cationic. If the POPG charges are also removed, the selectivity becomes only slightly cationic, and the total conductance is even lower. Turning off the charges in the POPG bilayer while retaining the arginine charges does not result in a significant difference in the total conductance or the current ratio. This suggests that in the presence of charged arginine residues, charges in the lipid bilayer do not have an appreciable direct effect on conductance characteristics in a formed pore, as the former clearly dominate all electrostatic behavior (however, the POPG charges clearly have a significant impact on the ability of the peptides to form a pore in the first place). Although complete removal of the arginine charges would not happen in any realistic physical situation, the results in this section show how drastic an effect these charges have.

In order to investigate the effects of individual arginine residues, we solved the PNP model for six additional situations, each corresponding to turning off the positive charges on the arginine residues for all peptides at a particular position. The calculations discussed were all performed on the structure corresponding to a snapshot at 93.5 ns of the NPT segment of the MD simulations of Langham and coworkers [17], using diffusion coefficient profile D4. The results are summarized in Table 4.

**Table 4:** Effects of turning off charges at various arginine positions.

Charge Removed	Conductance (pS) at +20 mV	I_Cl−_/I_K+_ at +20 mV
None	367	338
Arg-1	332	289
Arg-4	204	94
Arg-9	225	172
Arg-10	260	244
Arg-11	271	252
Arg-18	231	135

All data correspond to the snapshot at 80 ns of the MD simulation, using diffusion coefficient profile D4.

As shown in Table 4, the most significant effect is attained by turning off the charge at arginine position 4, which results in the largest drop in the conductance as well as the selectivity. This corroborates the hypothesis that the arginine residues at position 4 interact more strongly with the pore interior, likely due to their physical location within the pore. As such, while the other arginine residues likely play a larger role in peptide insertion, it would seem that the purpose of arginine-4 is primarily to effect ion passage through the pore by attracting chloride ions. However, it should be noted that the data in Table 4 represent only the conductance characteristics of one particular snapshot. Nonetheless, considering the consistency with the findings in the section ‘*Specific interactions of arginine side chains*’, this appears to be a worthwhile hypothesis that warrants further future investigation.

### Validity of PNP Equations

Since the characteristic dimensions of a typical transmembrane pore are commensurate with the size of ions, it behooves us to discuss the validity of a continuum theory in studying such systems. The PNP theory has been shown to overestimate channel conductance in an OmpF porin model by approximately 50% relative to a more rigorous Brownian dynamics (BD) approach [32]. Considering that the narrowest constriction in an OmpF porin channel has an area of 15 Å^2^, or an effective diameter of ∼4.4 Å [33], this agreement is surprisingly good for a theory that treats ions in a purely continuum representation. In another study designed to test the validity of the PNP theory, Corry and coworkers found that conductance values computed by PNP theory and BD simulations become equivalent for cylindrical pores with a diameter of 32 Å, with PNP results improving in charged channels [20]. Additionally, several studies have used PNP theory to model ion conduction through the α-hemolysin channel, which, at its narrowest constriction point, has an open cross-section with a diameter of approximately 15 Å [28]. Noskov and coworkers [28] as well as Dyrka and coworkers [29] found that the PNP theory overestimated experimental channel conductance values by approximately 30–60%. Furini and coworkers applied PNP modeling to a KcsA channel, which has a mean diameter of 2.8 Å in one region and 10–14 Å in another [30]. They obtained good agreement with experimental results, albeit by using an adjusted diffusion coefficient. The narrowest constriction point of the protegrin channel that we are interested in has a diameter ranging from 6–15 Å, depending on the conformation used. Considering the range of applications described above that have employed PNP theory and met with reasonable success, we believe the protegrin pore is a good candidate for such a study. More rigorous approaches such as Brownian dynamics or molecular dynamics with applied voltages are far more computationally demanding (particularly the latter), and would not have allowed us to explore an extensive parameter space.

We have applied the PNP theory to predict the conductance characteristics of several variations of a protegrin pore structure. We found that the best agreement with the experimentally measured net conductance was obtained using structures extracted from the latter part of molecular dynamics simulations of Langham and coworkers. Even considering variations in structures due to thermal fluctuations, all of these structures yielded conductance values near the experimental upper bound of 360 pS. The structure of the pore as surmised from NMR experiments has a larger opening at its narrowest constriction point than the structures from MD simulations, primarily due to differences in the orientation of amino acid side chains in the pore interior. PNP models based on this NMR structure yielded conductance values that are significantly higher than the experimentally measured values, even considering the expected typical errors arising from PNP theory. This suggests that the narrower structures observed in molecular dynamics simulations may be more realistic. In all of the structures tested, we observed an extremely high anion selectivity, which is to be expected for such a cationic pore, but surprisingly disagrees with the experimental findings of Sokolov and coworkers [19]. No significant voltage dependence of the conductance was observed in any of our models, corroborating the hypothesis that the voltage-dependence observed by Sokolov and coworkers [19] is a result of voltage-dependent channel formation, rather than an inherent feature of the protegrin pore structure. Due to the high sensitivity of PNP calculations to the diffusion coefficient profile, we have tested several different alternatives. We found that a diffusion coefficient set to 10% of the bulk value inside the channel yielded the best agreement with experimental data; this value therefore effectively represents a fitted parameter that accounts for several shortcomings in the mean field model. Even with these and other limitations of PNP theory in mind, we believe the general trends observed herein form a useful connection between the structural features and conductance characteristics of protegrin pores.

## Methods

This section outlines the methods employed in the modeling presented above. For methodological details related to the molecular dynamics simulations, the reader is referred to Langham et al [17].

### Poisson-Nernst-Planck Theory

In the PNP theory, ion flux **J** *_i_* is modeled according to the Nernst-Planck equation, which includes a Fickian diffusion term and a drift term to account for the effects of the electrostatic field:
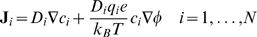
(3)Where

The index *i* corresponds to each of N diffusing ionic species
C*_i_* is the concentration of species *i*
ϕ is the electrostatic potential
D_i_ is the (space-dependent) diffusion coefficient of species *i*
q_i_ is the charge of species *i*
e is the elementary charge, 1.60217×10^−19^ C
k_B_ is Boltzmann's constant, 1.38065×10^−23^ J/K
T is the temperature, set to 310 K for all cases

At steady state conditions, mass continuity yields:
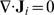
(4)Substituting equation (3) above for the species flux **J** *_i_* yields:
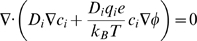
(5)This is the steady-state Nernst-Planck equation, which determines ion concentrations given an electrostatic potential field.

The electrostatic potential field, ϕ, depends on any fixed charges arising from the protein channel as well as the mobile charge arising from the space-dependent ion concentrations through the Poisson equation:
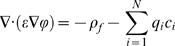
(6)Here, ρ_f_ represents the fixed charge density and ε is the (space-dependent) dielectric constant.

In the present work, we solve the coupled equations (5) and (6) numerically to obtain the steady-state ion concentrations and electrostatic potential. The electrical current across the pore can subsequently be calculated as:
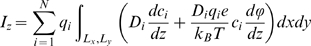
(7)Equation (7) above can be applied at any z-position along the pore axis, and shows only minor differences in the current values *I_z_* due to numerical inaccuracies. In most cases presented here, these variations are on the order of ∼2%.

The coupled PNP system (equations 5 and 6) is solved on a three-dimensional domain defined by the protegrin pore structure. The fixed charge density ρ_f_ is determined from the positions and charges of the protein and lipid atoms in the pore structure. The boundary conditions in the plane of the bilayer are periodic for all ion concentrations as well as for the electrostatic potential. In the direction perpendicular to the bilayer, the electrostatic potential is set to zero far away from the pore on the side of the protegrin β-sheet turns, and to the applied voltage (V_applied_) far away from the pore on the opposing side, nearest to the protegrin termini (refer to Figure 1). Similarly, ion concentrations are set to their bulk values far away from the pore in the direction perpendicular to the bilayer. Additionally, there is a no-flux boundary surrounding the peptide and lipid atoms that prevents ions from penetrating through the region occupied by the peptides and lipids. This can be physically interpreted as an approximate way to account for the short-range van der Waals repulsion between ions and peptide or lipid atoms.

Throughout the remainder of this manuscript, the z-direction will refer to the direction along the axis of the channel, perpendicular to the plane of the bilayer. The applied voltage values correspond to the value of the potential on the side of the pore nearest to the termini of the peptides. Letting L_x_, L_y_ and L_z_ represent the length of the computational domain, we can summarize the above boundary conditions as:
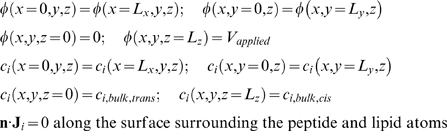
(8)


### System Setup

The charge density ρ_f_ was obtained by assigning to each atom location a point charge corresponding to the partial charge of that atom in the charmm27 force field [34]. Charges were distributed to the nearest grid points using a trilinear interpolation scheme. The ion accessibility region was defined by excluding all of the space occupied by peptide or lipid atoms. This space consists of a sphere with a radius equal to the sum of the atom's van der Waals radius (as given in the charmm27 force field) and the probe radius. For all of the results presented in the current work, a value of 1.4 Å was used for the probe radius, roughly corresponding to the effective radius of a water molecule. Probe radii corresponding to the radii of the ions were also tested and found to have relatively minor effects on conductance characteristics. The dielectric constant profile was obtained by assigning an appropriate value to the space surrounding each atom. Values of ε = 2 were assigned to lipid tail atoms, and values of ε = 5 were assigned to peptide and lipid head group atoms. Regions not occupied by any of the channel's atoms were assumed to be occupied by water, and assigned a relative dielectric constant of ε = 78. A few other schemes of assigning the dielectric constant were also tested, and found to have relatively minor effects on overall conductance characteristics (data not shown). In all cases, the dielectric constant profile was numerically smoothed with a Gaussian convolution filter of width σ = 1.5 Å. This improved numerical stability, and likely corresponds to a more realistic physical situation. Conductance characteristics were found to be insensitive to the choice of the smoothing parameter within a reasonable range (data not shown).

Four different methods were used to assign the space-dependent diffusion coefficient, as mentioned in the Results section. The resulting diffusion coefficient profiles were dubbed D1–D4, shown in Figure 6. Profile D1 consists simply of assigning the bulk literature value in all regions of the channel for both ions. These are 0.203 Å^2^/ps for chloride and 0.196 Å^2^/ps for potassium [35]. Since ion diffusion coefficients are likely to change in confined geometries [25], this profile likely represents a generous upper bound estimate.

Profile D2 is based on measuring the diffusion coefficient of chloride and sodium ions from molecular dynamics simulations carried out by Langham et al [17]; however, as already mentioned, there were only 2 events of sodium ions crossing the pore, and the sampling was simply not adequate to obtain reliable diffusion coefficient measurements inside the channel. Furthermore, the TIP3P water model [36] along with the charmm27 force field [34] used in the simulations is known to overestimate the diffusion coefficient of water by a factor of 2 [37]; as such, direct measurements of the diffusion coefficients of ions is not likely to yield accurate results. Instead, we looked at the approximate scaling of the diffusion coefficients in the MD simulations as compared to the measured bulk value, and applied the same scaling to the literature bulk value to create profile D2 for both ions. This amounts to a smooth decrease in the diffusion coefficient from the bulk value at the entrance of the channel to approximately 1/3 of the bulk value at the channel's centre.

Profile D3 is based on considering hydrodynamic effects of narrow constrictions on spherical particles. Based on the work of Paine and Scherr [27], Noskov et al [28] used the following correlation to calculate the diffusion coefficient of ions as a function of channel radius:
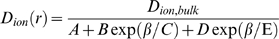
(9)Where β = R_ion_/R_pore_, and the empirical fitting parameters have the values given in Noskov et al (A = 0.64309, B = 0.00044, C = 0.06894, D = 0.35647, E = 0.19409). To ion radii used in our model are the van der Waals radii specified in the charmm27 force field (R_Cl−_ = 2.27 Å, R_K+_ = 1.764 Å), and the radius of the pore is calculated as an effective radius corresponding to the cross-sectional area found from a grid search. The resulting diffusion coefficient profiles for a snapshot at 93.5 ns are shown in Figure 6 (profile D3).

Profile D4 corresponds to setting the diffusion coefficients of both ions to their bulk literature values outside the channel, and to 10% of these values inside the channel. For chloride, this translates to a diffusion coefficient of 0.203 Å^2^/ps in the bulk region, and 0.0203 Å^2^/ps in the channel region. For potassium, the diffusion coefficient is 0.196 Å^2^/ps in the bulk region, and 0.0196 Å^2^/ps in the channel region. The channel region here is defined as the ion-accessible region bounded at 25 Å above and below the centre of the channel (i.e. −25<z<25, with z = 0 corresponding to the channel centre). Similar scaling was used by [31] and [30] in their PNP modeling of gramicidin S and KcSA, respectively, and was found to yield good agreement with experimental results. Although 10% of the bulk value likely underestimates the diffusion coefficient of ions inside the channel [26], given the success of this scaling, it may well be that it represents an effective correction of the tendency of PNP theory to overestimate ionic currents due to overestimation of the screening effects [20].

### Numerical Algorithm

The numerical algorithm used in the present work closely follows the finite difference (FD) method developed by Im and Roux [32] based on the algorithm proposed by Kurnikova et al [31]. In fact, the code we employed for the PNP solver is built closely on the main solver module of the 3d-PNP code provided on the website of Dr. Benoit Roux: http://thallium.bsd.uchicago.edu/RouxLab/pbpnp.html

Briefly, the numerical scheme consists of first converting the Nernst-Planck equation (5) to a Laplace-like form with the substitution
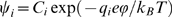
(10)This is known as a Slotboom transformation [38], and converts the Nernst-Planck equations to the form:
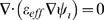
(11)Where 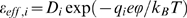


The finite difference representation of equation 11 above leads to the following expression for a given point in the finite difference grid (corresponding to the index 0) in terms of its neighbours (corresponding to the index j = 1,..,6).
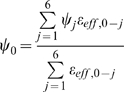
(12)where 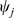 represents the effective concentration (vis. eq. 10) at neighbouring point j, and 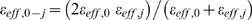.

The no-flux boundary condition is implemented by setting the value of 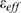 to zero for all points in the ion-inaccessible region. This has the same effect as setting the components of the flux perpendicular to the bounding region of the ion-inaccessible surface to zero. Ion concentrations are also initially set to 0 inside the ion-inaccessible region, and do not change due to the implementation of the no-flux boundary condition.

Similarly, the Poisson equation (6) has the following finite-difference representation of the central point in terms of its neighbours:
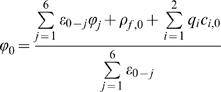
(13)Here, the summation over the index *j* represents summation over neighbouring grid points, while summation over *i* refers to summation over the ionic species, in this case only potassium and chloride. In equation (13) above, 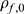 refers to the fixed charge density, and 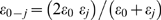.

Equations (12) and (13) above form the basis of successive overrelaxation (SOR) schemes for solving the Nernst-Planck and Poisson equations, respectively. The Poisson equation is solved first, using either zero everywhere or the solution of the linearized Poisson-Boltzmann equation as an initial guess for the potential. The computed electrostatic potential is then used to construct the effective concentration ψ_i_ and ε_eff_ profiles (see equations (10) and (11)), and this alternate form of the Nernst-Planck equation is solved using the FD scheme above (equation 12). The ion concentrations are then passed back to the Poisson equation, and a new potential is computed. The process is repeated until convergence of both equations is obtained.

In order to achieve stability of the solver, it is necessary to mix the solution from any given iteration of the Poisson equation with the solution from the previous iteration:
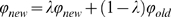
(14)Here, *ϕ_new_* represents the electrostatic potential computed at a given iteration of the Poisson equation, and *ϕ_old_* represents the potential at the previous iteration. The mixing parameter λ is typically set to 0.03, and increased during the iterative process whenever the concentration profiles do not change appreciably from one iteration of the Nernst-Planck equation to the next. Additionally, due to the high charge of the protegrin pore, we occasionally encountered numerical instabilities early on in the solution process. We employed a continuation strategy based on solving the equations to a low tolerance with a scaled down charge density or a high dielectric constant, then gradually increasing the charge density or decreasing the dielectric constant to their real values after achieving convergence at each intermediate value.

Numerical tolerances were typically set to 10^−14^ Å^−3^, or 1.7×10^−11^ M for the concentrations, and 10^−8^ e^−^/Å, or 1.4×10^−4^ mV for the electrostatic potential. The grid resolution used for all runs was 0.5 Å/grid point. A difference of around 17% in the measured current was observed between a resolution of 1.0 Å/grid point and the 0.5 Å/grid point resolution. Although we did not explore finer grid resolutions, we do not expect any significant impact on the results to arise from higher resolution grids. The measured conductance characteristics were found to be insensitive to dimensions larger than 115×115 Å in the bilayer plane, and 80 Å in the direction perpendicular to the bilayer (corresponding to a buffer of ∼15 Å of water on each side of the bilayer patch).

## Supporting Information

Text S1 Supporting Information
(3.57 MB DOC)
