# Supplementary figures and images for: Poisson-Nernst-Planck Models of Nonequilibrium Ion Electrodiffusion through a Protegrin Transmembrane Pore
Source: PLoS Comput Biol. 2009 Jan 30;5(1):e1000277. doi: 10.1371/journal.pcbi.1000277 (PMC2614469; doi:10.1371/journal.pcbi.1000277)

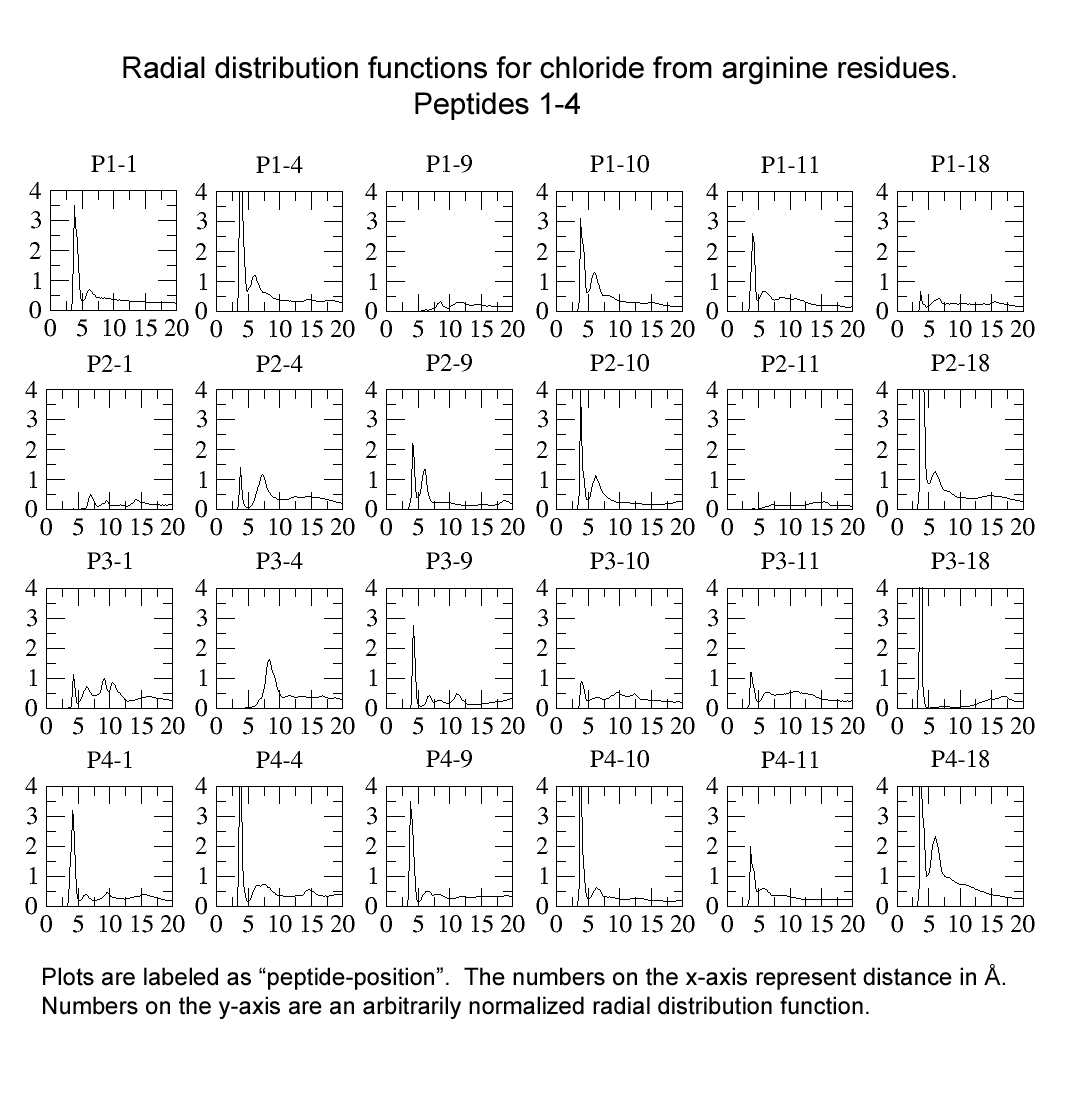

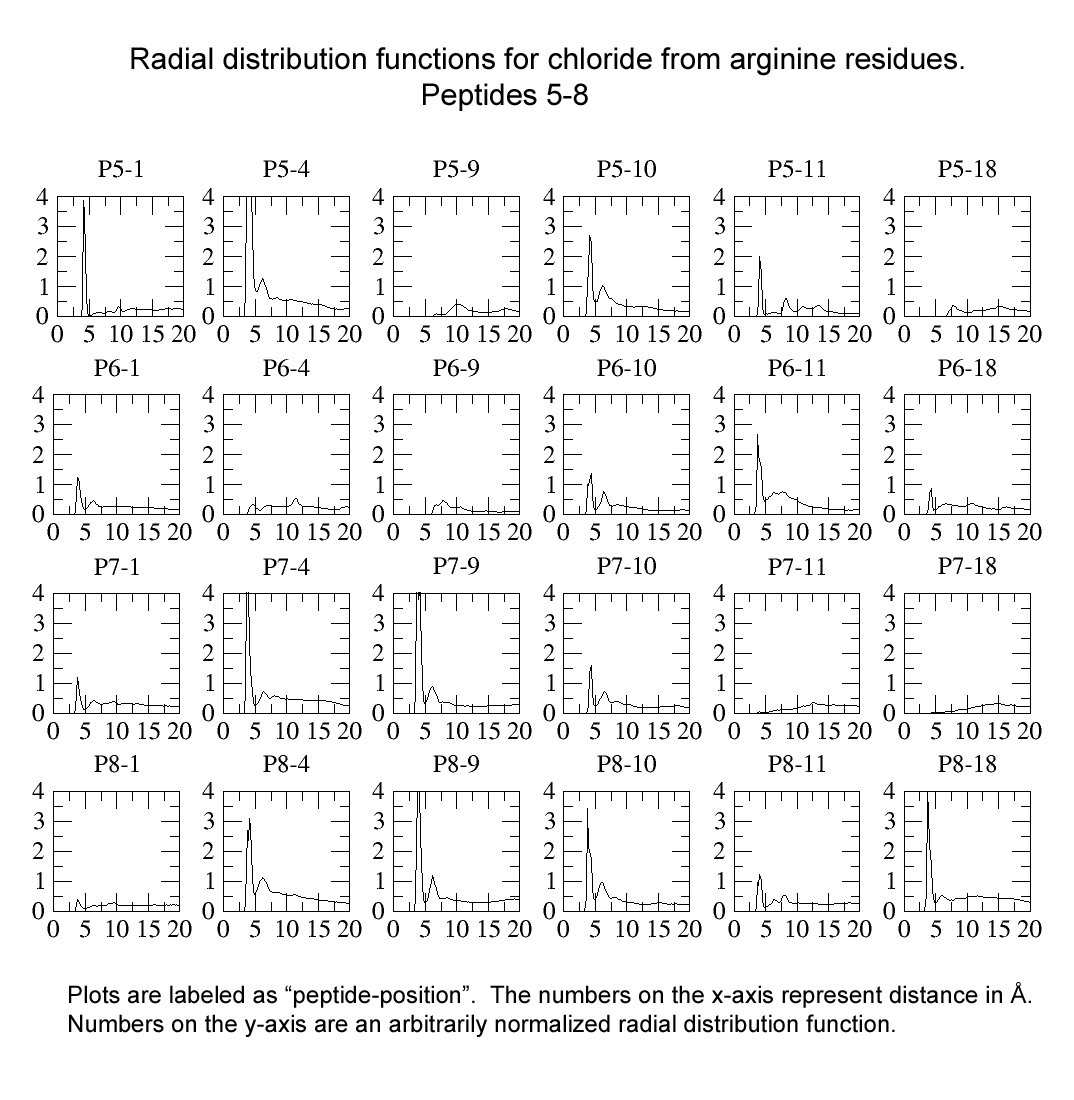


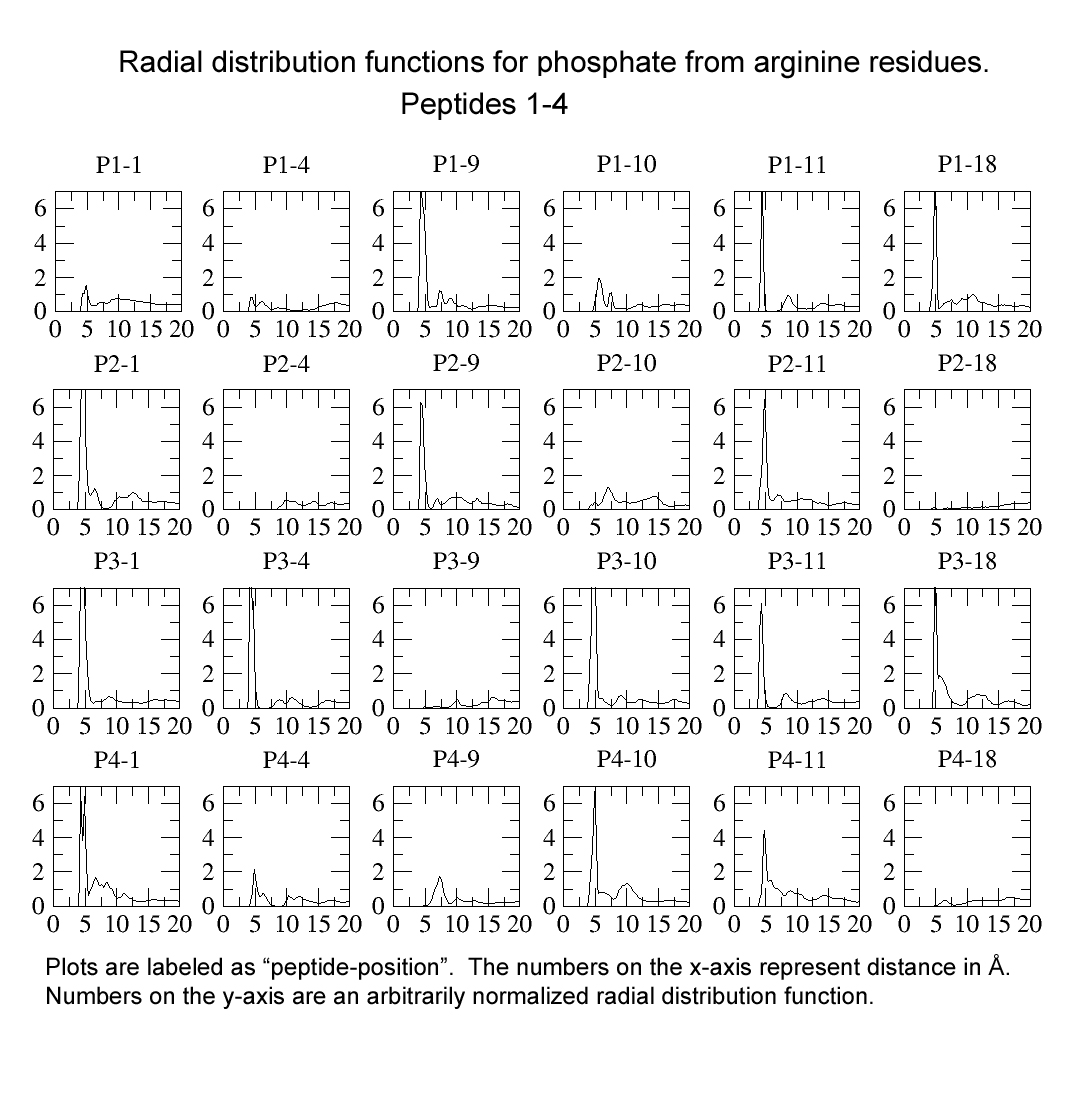

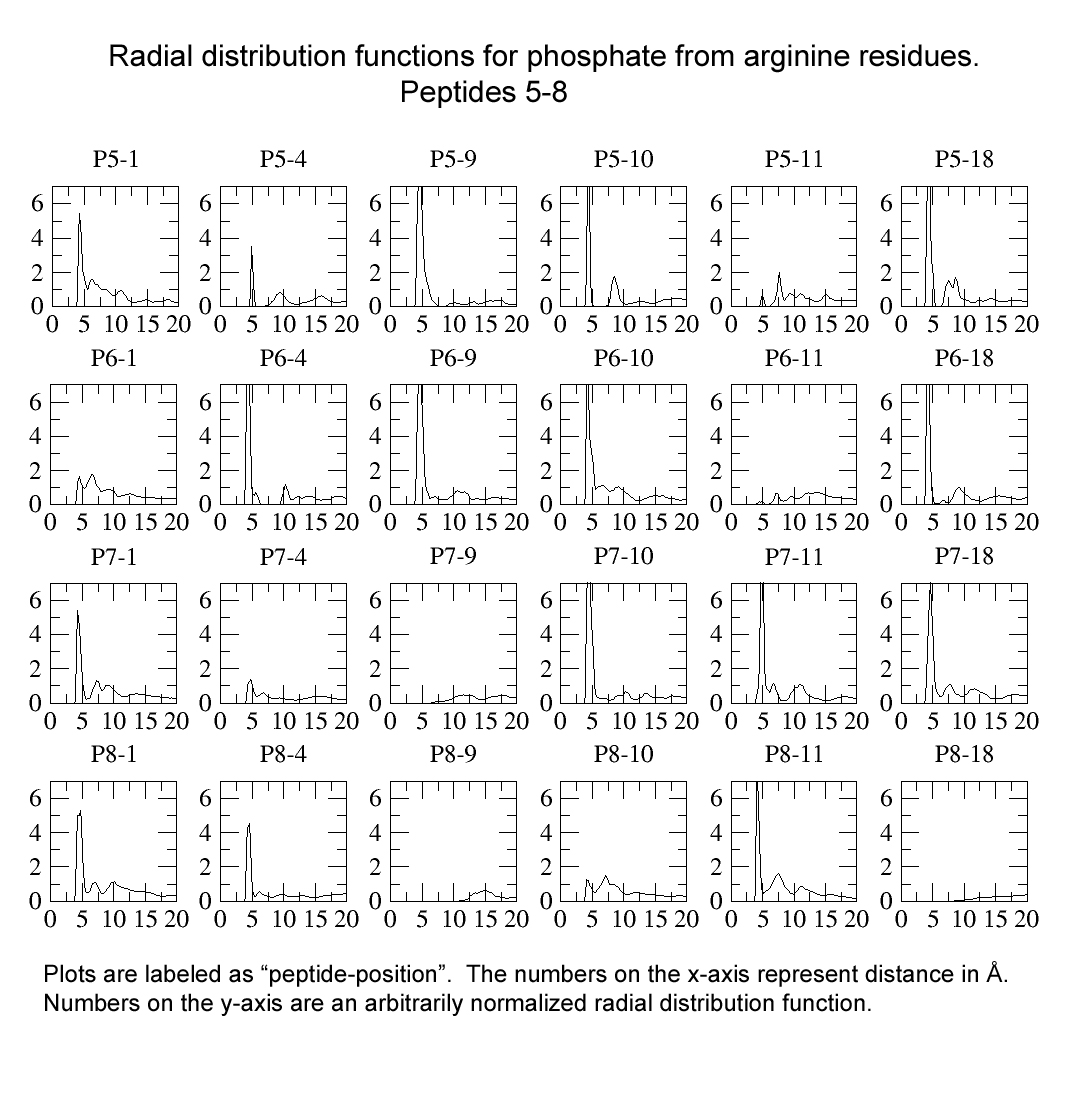


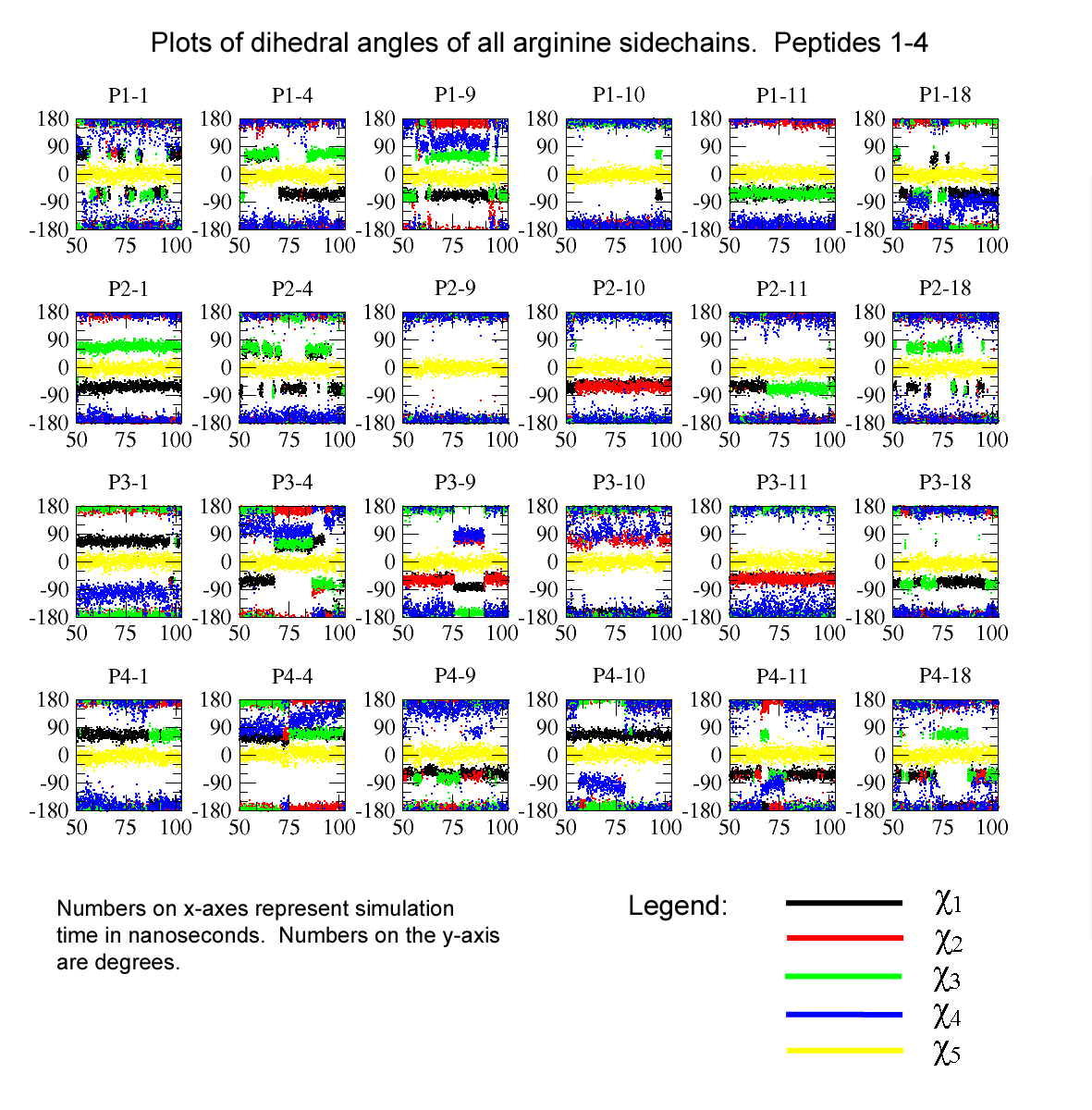


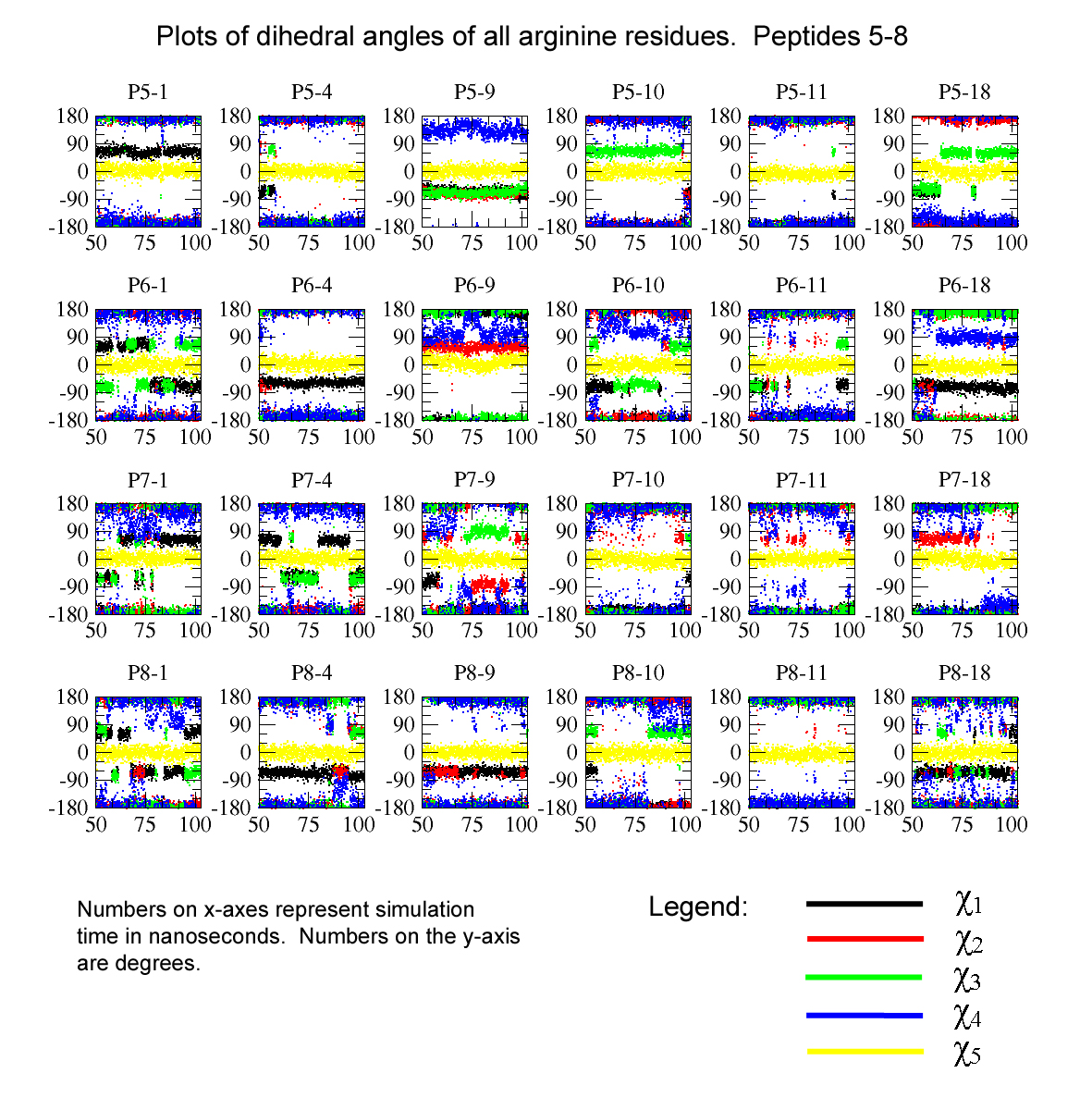

Supplement: Text S1 — Supporting Information (3.57 MB DOC) [file pcbi.1000277.s001.doc]
